# High-risk group and functional subtypes of non-suicidal self-injury in young adults with mental disorders

**DOI:** 10.3389/fpsyt.2023.1098178

**Published:** 2023-02-23

**Authors:** Huiru Yan, Yuyanan Zhang, Zhe Lu, Mingzhu Li, Yuqi Ge, Dongli Mei, Zhewei Kang, Yaoyao Sun, Qianqian Li, Hao Yan, Lei Yang, Peihua Song, Chuan Shi, Shaomei Shang, Weihua Yue

**Affiliations:** ^1^School of Nursing & Sixth Hospital, Peking University, Beijing, China; ^2^Research Unit of Diagnosis and Treatment of Mood Cognitive Disorder (2018RU006), Peking University Sixth Hospital, Institute of Mental Health, Chinese Academy of Medical Sciences, Beijing, China; ^3^NHC Key Laboratory of Mental Health, National Clinical Research Center for Mental Disorders (Peking University Sixth Hospital), Beijing, China; ^4^PKU-IDG/McGovern Institute for Brain Research, Peking University, Beijing, China; ^5^Chinese Institute for Brain Research, Beijing, China

**Keywords:** non-suicidal self-injury, mental disorder, young adult, risk factor, cluster analysis

## Abstract

**Background:**

Identifying high-risk groups of non-suicidal self-injury (NSSI) with multiple risk factors and different functional subtypes contribute to implementing person-centered interventions.

**Methods:**

We investigated NSSI profiles among a sample of 258 psychiatric inpatients aged 18–25 years. All participants completed well-validated measures of internal personal and external environmental characteristics. One-hundred and ninety patients reported a lifetime history of NSSI and completed an additional NSSI assessment. A k-means cluster analysis was conducted to extract characteristics of risk factors and functional subtypes. Independent sample *t*-test, analysis of variance and χ*^2^* test were used to test the difference of demographic statistical factors, risk factors and functional scores among groups with different frequency of NSSI.

**Results:**

The clustering of risk factors analyses supported 4-clusters. The proportion of repeat NSSI patients was the highest (67.1%) in the group with unfavorable personal and unfavorable environmental characteristics. Functional subtype clustering analyses supported 5-clusters. Among patients with repeated NSSI, those with depression were mainly accompanied by the “Sensation Seeking” subtype (39.7%), bipolar disorder mainly supported the “Anti-suicide” subtype (37.9%), and eating disorders were mostly “Social Influence” subtype (33.3%). There was an interaction between functional subtypes and mental disorders.

**Limitations:**

All participants were in treatment in a psychiatric service and the results may not be generalizable to a community sample. The data included retrospective self-report which may be inaccurate due to recall bias.

**Conclusion:**

It is necessary to identify high-risk groups of NSSI who with unfavorable personal and environmental characteristics and clinical interventions need to consider the heterogeneity of patients’ functional subtypes of NSSI.

## 1. Introduction

Non-suicidal self-injury (NSSI) refers to direct, deliberate harm of one’s own body without intent to die which is not socially sanctioned (1, 2). The Diagnostic and Statistical Manual of Mental Disorders Fifth Edition (DSM-5) suggested that it should be regarded as an independent diagnosis, called for further research, and reached the recommended diagnostic criteria through expert consensus. NSSI tends to occur in adolescents and young adults, and the incidence decreases in adulthood (3). In the past 10 years, the detection rate of NSSI has increased significantly in many countries (4–6). A longitudinal study showed that NSSI increased the risk of individual mental disorders, substance abuse, and self-harm, and led to serious damage to social functions (7). In addition, NSSI is also an independent predictor of suicide attempts (8). A meta-analysis based on longitudinal follow-up studies showed that the most common outcome of individuals with self-injurious thoughts and behaviors during the 4–5-year follow-up was suicide attempt (47.8%), followed by death (40.5%), and suicide ideation (11.6%) (9).

As for people with mental disorders, NSSI needs to attract greater attention. The prevalence of NSSI in clinical adolescent sample was as high as 40–70% (10, 11), and the risk of NSSI in psychiatric illnesses was significantly higher than that of physical illnesses (12). In a sample of 432 youth with bipolar disorder (BP), the lifetime prevalence was 34–37%, and the prevalence was 22% during the most recent mood episode (13, 14). The lifetime NSSI in adolescent patients with major depressive disorder (MDD) was 33–55% (8, 15). The weighted lifetime prevalence of NSSI in patients with eating disorders (ED) was 27.3%, including 21.8% for anorexia nervosa (AN) and 32.7% for bulimia nervosa (BN) (16). While patients with other mental diseases had a high risk of self-injury, including substance misuse or dependence, personality disorders, and schizophrenia (17). It can be seen from this that there is significant comorbidity between NSSI and mental illness. A meta-analysis showed that prior history of NSSI, cluster b personality, and hopelessness yielded the strongest effects (ORs > 3.0) among specific NSSI risk factors (18). In addition, other risk factors suggested by previous studies also include diagnosis of mental disorders, bad living environment in childhood, impaired family function, negative life events, etc. (11, 18, 19).

Considering the increasing prevalence of NSSI, the significant correlation with mental disorders, and its serious adverse effects, it is necessary for us to understand the clinical characteristics and risk factors of NSSI in people with mental disorders, to provide clues for the identification of people with a high risk of NSSI.

Besides, there is another key issue that needs our attention. As we all know, NSSI has many different functions, such as emotion regulation, social influence, anti-dissociation, anti-suicide, self-punishment, sensation seeking, etc. (20). In order to better understand the prevalence of NSSI functions and provide key information to address the needs of different NSSI populations, Taylor et al. (2) conducted a systematic review and meta-analysis and found that intrapersonal functions were the most common function (66–81%) including emotion regulation, while the interpersonal functions were less common (33–56%). There was different prevalence of NSSI functions in different samples such as community adolescents and clinical patients (2). Patients with different functions also have different needs for support and intervention. For example, the key of dialectical behavior therapy (DBT) and emotion regulation group therapy (ERGT) is to improve emotional tolerance and regulation function. For individuals whose NSSI function is mainly social influence or sensation-seeking, ERGT is not the preferred intervention. A study explored the sub-group profiles of NSSI in a community sample of young adults and found that NSSI in distinct sub-groups had heterogeneity (21). However, few existing studies have explored different NSSI functional heterogeneity in diverse groups. Thus, a full understanding of the functional characteristics of NSSI in different groups is conducive to providing valuable information for the prevention and treatment of NSSI.

The present study aimed to promote the identification of high-risk groups of NSSI in mental disorder (such as depression, BP and ED), and explore the functional subtypes of NSSI. The first research objective was to investigate the clinical characteristics and risk factors (including personal and environmental characteristics). Then we tested the association between multiple risk factors and frequency of NSSI to identify the high-risk groups. The second research objective was to examine whether NSSI patients with common mental disorders have functional subtypes of NSSI and to associate those subtypes with the diagnosis. And we further explored the functional preferences among different mental disorders. We hope to provide ideas and information for early prevention, and person-centered intervention toward NSSI patients through the above investigation and analysis.

## 2. Materials and methods

### 2.1. Participants and procedures

Participants (16–25 years old) were consecutively admitted to a psychiatric hospital in Beijing, China from July 2021 to March 2022. Inclusion criteria: a. individuals conform with the diagnostic criteria of depressive episode or recurrent depressive disorder, BP and ED in ICD-10 which were interviewed and diagnosed by two senior doctors in the ward. b. individuals obtain the written informed consent of the patient or guardian. Exclusion criteria: a. individuals have simple suicidal behavior and do not meet the diagnostic criteria of NSSI. b. individuals suffer from developmental delay. c. individuals suffer from severe unstable physical diseases.

After strict screening, 275 cases signed informed consent and were included in the group. 258 valid questionnaires were collected and the effective response rate was 93.8%. This study has been approved by the biomedical ethics committee of the Sixth Hospital of Peking University.

### 2.2. Measurements

The basic characteristics of participants were collected through the case report form (CRF), including demographic data, past medical history and accompanying diseases, and clinical information about existing mental disorders. In addition to the CRF, the following measures were administered. We evaluated the internal personal characteristics of patients through the Self-Rating Anxiety Scale (SAS), the Self-Rating Depression Scale (SDS), the NEO Five-Factor Inventory (NEOFFI) and the Chinese Internet Addiction Scale’s revision (CIAS-R). The external environmental characteristics were evaluated by the Adolescent Self-Rating Life Events Check List (ASLEC), the Childhood Trauma Questionnaire (CTQ), the Family Assessment Device (FAD) and the Social Support Rate Scale (SSRS).

#### 2.2.1. Ottawa Self-Injury Inventory (OSI)

Ottawa Self-Injury Inventory was developed by Cloutier and Nixon, which translated and revised into Chinese by Zhang Fang of Shanghai Mental Health Center (22). OSI is used to evaluate the frequency of recent NSSI thoughts and behaviors, the initial and continuous functions of NSSI, addiction characteristics. The functions of NSSI are assessed by 27 items (e.g., to relieve nervousness/fearfulness) with a range response option from 0 (never) to 4 (always) which is a separate subscale named Ottawa Self-Injury Inventory-Function (OSI-F). The OSI has excellent internal consistency scores of 0.64 to 0.95 within a Chinese sample of adolescents and is a valid and reliable instrument.

#### 2.2.2. Self-Rating Anxiety Scale (SAS)

The SAS (23) contains 20 items with a range response option from 1 (no or little time) to 4 (most or all the time). Higher scores indicate higher levels of anxiety. A total score of less than 50 is normal, 50–59 is mild anxiety, 60–69 is moderate anxiety, and more than 69 is severe anxiety.

#### 2.2.3. Self-Rating Depression Scale (SDS)

The SDS (23) contains 20 items with a range response option from 1 (no or little time) to 4 (most or all the time). Higher scores indicate higher levels of depression. A total score of less than 53 is normal, 53–62 is mild depression, 63–72 is moderate depression, and more than 73 is severe depression.

#### 2.2.4. NEO Five-Factor Inventory (NEOFFI)

The NEOFFI (24) contains 60 items with a range response option from 1 (strongly disagree) to 5 (strongly agree), and is used to assess the five broad personalities including Neuroticism, Extraversion, Openness to experience, Agreeableness, and Conscientiousness. The NEOFFI is valid and reliable with excellent internal consistency scores of 0.82 to 0.89 in Chinese sample.

#### 2.2.5. Chinese Internet Addiction Scale’s revision (CIAS-R)

The CIAS-R (25) consists of 19 items, and evaluates the symptoms of Internet addiction (Internet addiction tolerance, compulsive Internet use and Internet addiction withdrawal symptoms) and problems related to Internet addiction (time management problems and interpersonal and health problems), with a range response option from 1 (strongly disagree) to 4 (most strongly agree). Higher scores indicate the greater the possibility and tendency of Internet addiction. A total score of less than 46 is normal, 46–53 is the Internet dependence group, and more than 53 is the Internet addiction group.

#### 2.2.6. Adolescent Self-Rating Life Events Check List (ASLEC)

The ASLEC (23) consists of 27 items. Participants were asked to answer whether listed events were happened in the past 12 months. If it has happened, the score will be selected according to the impact of the event, ranging from 1 (no impact) to 5 (extremely severe impact). Higher scores indicate the severer impact of negative life events. The ASLEC is valid and reliable with excellent internal consistency scores of 0.85 in Chinese sample of adolescents.

#### 2.2.7. Childhood Trauma Questionnaire (CTQ)

The CTQ (26) is used to investigate the growth experience of childhood (before the age of 16). The Chinese version has a total of 28 items, including 5 subscales of emotional mistreatment, physical mistreatment, sexual abuse, emotional negligence, physical negligence. The scale adopts a range response option from 0 (never) to 4 (always). The CTQ is valid and reliable with internal consistency scores of 0.64 in Chinese sample of adolescents.

#### 2.2.8. Family Assessment Device (FAD)

The FAD (23) consists of 60 items and evaluates seven dimensions: problem solving, communication, roles, affective responsiveness, affective involvement, behavior control, general functioning. The scale adopts a range response option from 1 (strongly disagree) to 4 (strongly agree). Higher scores indicate the unhealthier functions of family. It The FAD has been shown to be valid and reliable.

#### 2.2.9. Social Support Rate Scale (SSRS)

The SSRS (23) consists of 10 items and evaluates three dimensions: objective support, subjective support, support utilization. The SSRS has been shown to be valid and reliable with test-retest reliability of 0.92. In order to facilitate the display in the figure, we adopt the reverse scoring method of SSRS. The full score of the scale minus the report score is the SSRS-reverse score (78 is the full score of the scale). The higher the SSRS-reverse is, the worse social support is.

### 2.3. Statistical analysis

Descriptive analyses were performed to describe the demographic characteristics of the sample. Independent sample *t*-test or *F*-test were used for continuous variables, and χ*^2^* test was used for classification variables, with *P* < 0.05 as the statistical significance threshold.

K-means is a clustering algorithm that uses distance as the similarity evaluation index. The closer the distance between two objects, the greater the similarity. K-means clustering algorithm believes that clusters are composed of close objects, so it will get compact and independent clusters as the goal. It has many advantages, such as fast and simple calculation, and its time complexity is close to linear (27).

We conducted cluster on two aspects of the analysis. First, the standardized values (*Z-score*) of SAS score, SDS score, Neuroticism score, CIAS-R score, ASLEC score, CTQ score, FAD score, and SSRS score-reverse were used as clustering components for analysis, to find out the specific multiple risk factors in the population with mental disorders. And χ*^2^* test was performed on the clustering center and NSSI frequency to identify the high-risk population of NSSI. The second cluster analysis was conducted with the standardized values (*Z-scores*) of four NSSI functions valued by OSI-F to explore different functional subtypes in NSSI patients. The best clustering number is determined by calculating the within groups sum of square errors (WSSE) and elbow method using R language. Then the K-means cluster analysis was conducted by SPSS 26.0.

## 3. Results

### 3.1. Sample characteristics and preliminary analyses

Among the sample of 258 participants, 74.8% (*n* = 193) were female, and 25.2% (*n* = 65) were male, with a mean age of 18.89 years (SD = 2.49). More than half of the samples (*n* = 138) were diagnosed with depressive disorder, 23.6% (*n* = 61) were BP, and 22.9% (*n* = 59) were ED. Repeated NSSI was defined as the individual who has 5 or more days, engaged in NSSI in the past year, which meets the recommended diagnostic criteria for NSSI in DSM-5. Occasional NSSI was defined as the individual engaged in NSSI 1 to 4 times in the past. Among the full sample, 73.6% (*n* = 190) reported a lifetime prevalence of NSSI, of which 58.9% (*n* = 112) were repeated NSSI. The proportion of repeated NSSI in patients with depression and BP was significantly higher than that in patients with ED, and the latter was mainly with occasional NSSI or without NSSI (*P* = 0.006). The repeated NSSI individuals had a younger average age (18.16 ± 1.97) than occasional/without NSSI individuals (19.45 ± 2.71). The same trend also existed in the onset age of mental disorder and the differences were all significant. Additionally, patients with suicidal ideation (SI) (*n* = 99, OR = 5.47, 95% CI = 2.81–10.63) and suicide attempts (SA) (*n* = 74, OR = 5.74, 95% CI = 3.34–9.85) in the repeated NSSI group were significantly more than those in the occasional/without NSSI group. See Table 1 for more details.

**TABLE 1:** Basic characteristics of participants [*N* (%), mean ± SD].

	Total	Repeated NSSI	Occasional/Without NSSI	*t*/χ^2^	*P*-value
	**(*N* = 258)**	**(*N* = 112)**	**(*N* = 146)**		
Age	18.89 ± 2.49	18.16 ± 1.97	19.45 ± 2.71	17.929	<0.001***
Onset age of mental disorder	15.96 ± 2.56	15.44 ± 2.19	16.37 ± 2.76	8.654	0.004**
Diagnosis				10.126	0.006**
Depressive disorder	138 (53.5)	68 (49.3)	70 (50.7)		
Bipolar disorder	61 (23.6)	29 (47.5)	32 (52.5)		
Eating disorder	59 (22.9)	15 (25.4)	44 (74.6)		
Gender				2.279	0.131
Male	65 (25.2)	23 (35.4)	42 (64.6)		
Female	193 (74.8)	89 (46.1)	104 (53.9)		
Suicidal ideation				28.21	<0.001***
Yes	184 (71.3)	99 (53.8)	85 (46.2)		
No	74 (28.7)	13 (17.6)	61 (82.4)		
Suicidal attempt				42.89	<0.001***
Yes	111 (45.1)	74 (66.7)	37 (33.3)		
No	147 (54.9)	38 (25.9)	109 (74.1)		

**P < 0.01, ***P < 0.001.

### 3.2. Characteristics of NSSI

Within the sub-sample of NSSI (*n* = 190), 67.9% injured “hand/fingers,” 66.4% injured “lower arm/wrist.” 38.8% of patients reported that the area most often injured was “lower arm or wrist.” Regarding the methods of NSSI, 61.2% used “cutting,” and more than half of the patients used “scratching,” “hitting” and “headbanging.” 30.2% of patients reported that the method most often used was “cutting.” The average number of injured areas and the methods of NSSI in the repeated NSSI group were significantly higher than those in the occasional NSSI group (see Supplementary Table 1). As for the addiction to NSSI, the score of patients in the repeated group was 13.03 ± 6.70, which was significantly higher than that in the occasional group (6.85 ± 5.66). See Supplementary Table 2 for more details.

### 3.3. Risk factors of NSSI and characteristics of high-risk groups

#### 3.3.1. Risk factors among different groups

In the investigation of the possible risk factors of NSSI, compared with the occasional/without NSSI group, patients in the repeated group reached the level of moderate anxiety (63.91 ± 15.16) and severe depression (73.34 ± 13.18), and reported the higher neuroticism scores (37.33 ± 6.36), the lower conscientiousness scores (28.42 ± 6.54), the severer negative life events (52.80 ± 22.01), the unhealthier family function (150.72 ± 22.96) and the poorer social support utilization (5.84 ± 1.73). The differences were all significant (see Supplementary Table 3).

#### 3.3.2. Cluster analysis-identification of NSSI high-risk groups

**FIGURE 1:**
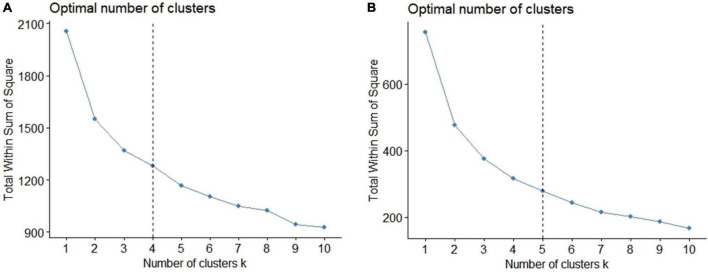
The elbow diagrams of total within sum of square errors (WSSE). **(A)** The cluster analysis of NSSI risk factors. The WSSE decline slope of the cluster number from 3 to 5 is almost the same, so solutions comprising of 3, 4, and 5 possible clusters were considered. **(B)** The cluster analysis of NSSI functions. The WSSE decline slope of the cluster number from 4 to 6 is almost the same, so solutions comprising of 4, 5, and 6 possible clusters were considered.

**FIGURE 2:**
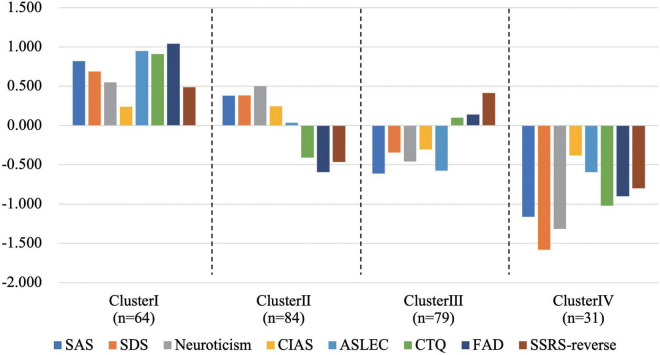
The four cluster centers of risk factors. Cluster I-Unfavorable Internal personal characteristics and Unfavorable External environmental characteristics (UIUE); Cluster II-Unfavorable Internal personal characteristics and Favorable External environmental characteristics (UIFE); Cluster III-Favorable Internal personal characteristics and Unfavorable External environmental characteristics (FIUE); Cluster IV- Favorable Internal personal characteristics and Favorable External environmental characteristics (FIFE).

In the elbow diagram, we focus on the rate of WSSE decline along with the increase of the number of clusters, that is, the slope. When WSSE declines very slowly, it is considered that further increasing the number of clusters will not enhance the clustering effect. As seen in Figure 1A, the WSSE decline slope of the cluster number from 3 to 5 is almost the same, so solutions comprising of 3, 4, and 5 possible clusters were considered. When the number of clusters was 3, the characteristics of each cluster center were too single to provide reference value to clinical application. When the number of clusters was 5, the characteristics of the two clustering centers were too similar, which affected the interpretability of the solution. When the number of clusters was 4, final clustering centers can reflect the differences between internal personal characteristics and external environmental characteristics well. Subsequently, the 4-cluster solution was selected, which is presented in Figure 2.

Cluster I was named “Unfavorable Internal personal characteristics and Unfavorable External environmental characteristics-UIUE” according to the significantly higher scores of personal internal characteristics (including SAS, SDS, Neuroticism and CIAS-R) and external environment characteristics (including ASLEC, CTQ, FAD and SSRS-reverse). Cluster II was named “Unfavorable Internal personal characteristics and Favorable External environmental characteristics-UIFE.” Cluster III was named “Favorable Internal personal characteristics and Unfavorable External environmental characteristics-FIUE.” Cluster IV was named “Favorable Internal personal characteristics and Favorable External environmental characteristics-FIFE.”

Among the Cluster I-UIUE, 67.1% of patients had repeated NSSI. Nearly half of patients in Cluster II-UIFE reported repeated NSSI (47.7%). Patients with occasional NSSI (39.2%) accounted for the largest proportion in Cluster III-FIUE, while patients without NSSI possessed the most proportion in Cluster IV-FIFE (58.1%) (see Supplementary Table 4). The NSSI frequency of each cluster center is statistically different, and there was a moderate correlation between cluster center and NSSI frequency (*c* = 0.364).

The association between cluster centers and frequency of NSSI was tested using a logistic regression model. Set dummy variables with Cluster IV-FIFE as reference. The results showed that Cluster I-UIUE and Cluster II-UIFE had a significant effect on repeated NSSI [*Exp(B)-* Cluster I = 10.648, *Exp(B)-* Cluster II = 4.727], while the effect of Cluster III-FIUE was not significant (see Supplementary Table 5). Individuals with unfavorable internal personal characteristics and unfavorable external environmental characteristics were high-risk groups for repeated NSSI.

### 3.4. The function of NSSI and the differences among subtypes

#### 3.4.1. Level of different functions of NSSI

According to the results of OSI-F factor analysis (see Supplementary Table 6), “Emotional regulation,” “Social influence,” “Sensation seeking,” and “Anti-suicide” were the four functions of NSSI in the sample of this study. The average score of emotion regulation was the highest (2.28 ± 1.02), followed by sensation seeking (1.52 ± 1.12), anti-suicide (1.12 ± 1.44), and the social influence was the lowest. No significant difference was found in functional scores between different genders. But the higher the NSSI frequency was, the higher the functional score was. In addition, there were significant differences in the scores of social influence and sensation seeking among different diagnoses of mental disorders (see Table 2). Further pairwise comparison results showed that the score of social influence in patients with eating disorder was significantly higher than that of patients with depressive disorder (*P*_*adjust*_ = 0.021), and the score of sensation seeking in patients with bipolar disorder was significantly higher than that of patients with eating disorders (*P_*adjust*_* = 0.042).

**TABLE 2:** The average function scores of participants with NSSI [mean ± SD].

	Emotion regulation	Social influence	Sensation seeking	Anti-suicide
Total	2.28 ± 1.02	0.81 ± 0.73	1.52 ± 1.12	1.12 ± 1.44
Gender				
Male	2.28 ± 1.14	0.87 ± 0.85	1.68 ± 1.26	1.30 ± 1.45
Female	2.29 ± 0.99	0.80 ± 0.70	1.47 ± 1.08	1.07 ± 1.44
*t*	−0.054	0.533	1.041	0.911
*P*-value	0.957	0.299	0.595	0.363
Frequency of NSSI				
Occasional	1.80 ± 1.04	0.73 ± 0.71	1.04 ± 0.95	0.85 ± 1.29
Repeated	2.62 ± 0.87	0.87 ± 0.74	1.85 ± 1.12	1.31 ± 1.52
*t*	−5.706	−1.309	−5.412	−2.256
*P*-value	<0.001***	0.192	<0.001***	0.025*
Diagnosis				
Depressive disorder	2.26 ± 1.02	0.73 ± 0.65	1.51 ± 1.10	1.11 ± 1.47
Bipolar disorder	2.38 ± 1.12	0.81 ± 0.79	1.80 ± 1.27	1.46 ± 1.52
Eating disorder	2.22 ± 0.87	1.09 ± 0.85	1.11 ± 0.84	0.66 ± 1.12
*F*	0.308	3.078	3.711	3.003
*P*-value	0.736	0.048*	0.026*	0.052

*P < 0.05; ***P < 0.001. The average function score was calculated by dividing the total score of items by the number of items.

#### 3.4.2. Cluster analysis-relationship between NSSI functional subtypes and mental disorders

**FIGURE 3:**
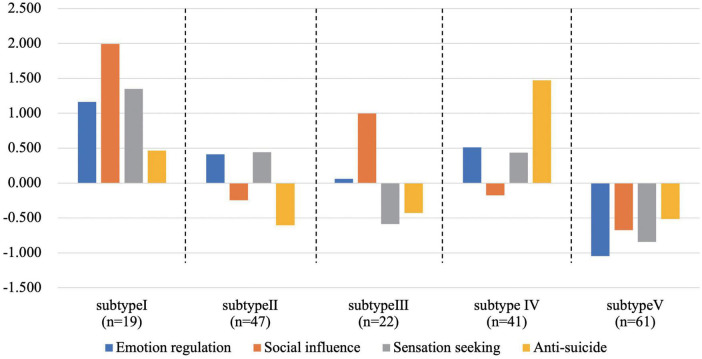
The functional subtypes of NSSI. All the four standardized score of NSSI function were higher than the average level in the subtype I; all the four standardized score of NSSI function were lower than the average level in the subtype V; the standardized score of “sensation seeking” was obviously higher than the average level compared with other functions in subtype II; the standardized score of “anti-suicide” was obviously higher than the average level compared with other functions in subtype III; the standardized score of “social influence” was obviously higher than the average level compared with other functions in subtype IV.

K-means cluster analysis was conducted with the Z-score of the four NSSI functions. In the elbow diagram in Figure 1B, the WSSE decline slope of the cluster number from 4 to 6 is almost the same, so solutions comprising of 4, 5, and 6 possible clusters were considered. When the number of clusters was 4, the features related to the “sensation seeking” function was not reflected in the cluster center. When the number of clusters was 6, two of the final cluster centers had similar features and did not have clinical significance. The result with 5-clusters was finally selected (see Figure 3). There were 5 subtypes of NSSI function according to the cluster analysis. Subtype I was named “Typical NSSI” because of the significantly higher scores of all four NSSI functions. Subtype II was named “Sensation Seeking NSSI” because of the significantly higher scores of sensation seeking. And it cannot be ignored that the emotion regulation also played an important role in this subtype according to its standardized score. The same naming method was applied to the other three subtypes. Subtype III was “Social Influence NSSI,” Subtype IV was “Anti-Suicide NSSI,” and Subtype V was “Untypical NSSI.”

Further analysis found that functional subtypes were correlated with NSSI frequency and mental disorders (see Table 3). There were 48.7% of patients with occasional NSSI divided into “Subtype V-Untypical NSSI,” while the proportion of patients with “Subtype II-Sensation Seeking NSSI” was higher in the repeated group (32.1%) (χ*^2^* = 28.740, *P* < 0.001). Interestingly, patients with “Subtype II-Sensation Seeking NSSI” had the highest proportion in depressive disorder (39.7%), and BP patients were mainly “Subtype IV – Anti-Suicide NSSI” (37.9%), while ED patients were mainly “Subtype III-Social Influence NSSI”. No similar results were observed in patients in the occasional NSSI group.

**TABLE 3:** The association between functional subtypes and diagnosis [*N* (%)].

	Subtype I	Subtype II	Subtype III	Subtype IV	Subtype V	χ*2*	*P*-value
	**(*n* = 19)**	**(*n* = 47)**	**(*n* = 22)**	**(*n* = 41)**	**(*n* = 61)**		
Occasional NSSI						10.936	0.205
Depressive disorder (*n* = 43)	3 (7.0%)	6 (14.0%)	6 (14.0%)	7 (16.3%)	21 (48.8%)		
Bipolar disorder (*n* = 18)	1 (5.6%)	2 (11.1%)	1 (5.6%)	4 (22.2%)	10 (55.6%)		
Eating disorder (*n* = 17)	1 (5.9%)	2 (11.8%)	7 (41.2%)	0 (0.0%)	7 (41.2%)		
Total (*n* = 78)	5 (6.4%)	10 (12.8%)	14 (17.9%)	11 (14.1%)	38 (48.7%)		
Repeated NSSI						28.768	<0.001***
Depressive disorder (*n* = 68)	5 (7.4%)	27 (39.7%)	3 (4.4%)	18 (26.5%)	15 (22.1%)		
Bipolar disorder (*n* = 29)	7 (24.1%)	7 (24.1%)	0 (0.0%)	11 (37.9%)	4 (13.8%)		
Eating disorder (*n* = 15)	2 (13.3%)	3 (20.0%)	5 (33.3%)	1 (6.7%)	4 (26.7%)		
Total (*n* = 112)	10 (8.9%)	36 (32.1%)	25 (22.4%)	12 (10.7%)	29 (25.9%)		

***P < 0.001. Subtype I-Typical NSSI. Subtype II-Sensation Seeking NSSI. Subtype III-Social Influence NSSI. Subtype IV-Anti-Suicide NSSI. Subtype V-Untypical NSSI.

## 4. Discussion

The current findings demonstrated that individuals with more risk factors were more likely to engage in repeated NSSI and patients with unfavorable internal personal characteristics and unfavorable environmental characteristics were the high-risk groups of NSSI. There were different functional subtypes of NSSI and the preferences of function among mental disorders were significant, that was, the functional subtypes interacted with diagnosis.

### 4.1. NSSI in hospitalized young adults with common mental disorders

In this study, it can be found that the prevalence of NSSI was high in young adult patients with emotional disorders and ED. Glenn et al. (28) had similar findings in hospitalized adolescents with mental disorders. According to the data of the Youth Risk Behavior Surveillance System (YRBSS) in 2015, 17.6% of the high school students had NSSI at least once in the past year (29). By comparison, the prevalence and severity of this behavior in clinical samples with mental disorders were much higher. The results of the current study provide references for understanding the situation of NSSI in mental disorders. Moreover, our results supported that patients with repeated NSSI had a five times higher risk of SI and SA than patients with occasional or without NSSI. NSSI is considered to be effective in resisting suicide in the short term (20). A previous study using ecological momentary assessment (EMA) real-time monitoring demonstrated that SI increased before the episode of NSSI, and then decreased. However, repeated NSSI may make individuals accustomed to fear of physical pain or injury, thus improving suicide capability according to the interpersonal theory of suicide (30). These findings highlight the importance of regarding NSSI as an important indicator of potential suicide risk.

### 4.2. High-risk group of NSSI

When patients had a high level of anxiety and depression, experienced more negative life events, childhood abuse experience, poor family function or social support, they were more likely to engaged in repeated NSSI. From the perspective of a single risk factor, previous studies have confirmed the significant correlation between NSSI and anxiety, which is similar between NSSI and depression (31, 32). Various environmental factors also have a great impact on NSSI such as stressful life events. In line with Nock’s model, life events might act as risk factors of NSSI both in the short run and forward (33, 34). In the short run, life events can cause dysfunction of the immune and stress-response systems and result in individual vulnerability to stressors (35, 36). In the long run, Kaess et al. (37) found that the number of life events predicted the onset of self-injury in the second year, which indicated that life events may have great influence on the development of self-injury. Poor family environment and social support are related to the maintenance of NSSI, while a better environment is related to the reduction of this behavior (38). Previous meta-analyses summarized various influencing factors of NSSI (39). It is worth mentioning that our clustering results showed that neuroticism and Internet addiction were also factors that distinguish different NSSI risk groups. Goddard, et al. (40) found that individuals in the cluster with high neuroticism and low scores of other personality traits, whose frequency of NSSI was significantly higher than that of other clusters. Besides, some studies suggested that the correlation between Internet addiction and NSSI, which was a risk factor for NSSI (41, 42).

Although many risk factors have been screened, we need to understand them from a comprehensive perspective. To substantially improve predictive power beyond single risk factors, it is necessary to combine diverse NSSI risk factors and consider in what ways they combine (e.g., additive, interaction). The clustering results of this study provide a reference in this field. The combination of risk factors was different between patients with repeated NSSI and patients with occasional or no NSSI. It was obvious that repeated NSSI was the most frequently reported among individuals with unfavorable personal and environmental characteristics. Even if the environmental characteristics were relatively favorable, repeated NSSI was easy to occur when individuals had unfavorable internal personal characteristics. By contrast, NSSI was less likely to occur when individuals had favorable personal and environmental characteristics at the same time. The characteristics of the two dimensions had different effect sizes on NSSI and there might be interaction between them. The diathesis-stress model pointed out that stress will transform the potential of susceptibility into the *status quo* of psychopathology on the basis of individual’s diathesis or vulnerability (43). In other words, the effects of stress are dependent on the diathesis. And the synergism between diathesis and stress exceeds their combined separate effects (43). Therefore, unfavorable personal characteristics were necessary condition for the occurrence of NSSI. Combined with environmental exposure factors, especially acute stress factors (such as companion injury, loss of interpersonal relationship, etc.) (44, 45), the occurrence of NSSI may increase. It suggests that we explore the interaction among different influencing factors of NSSI, and further identify NSSI high-risk groups with combinations of certain factors, to carry out early prevention, or targeted intervention according to the characteristics of the group.

### 4.3. Functional subtypes of NSSI

In current research sample, the main function of NSSI was emotion regulation, followed by sensation seeking and anti-suicide, and finally social influence. Although there are different functional theoretical models, such as Klonsky (20) and Nock (34), the most common functions of NSSI are still emotion regulation, social influence and so on. The meta-analysis of Taylor, et al. (2) also showed that 66–81% of patients engaged in NSSI for the purpose of self-reinforcement, including 63–78% of patients involved in emotion regulation, and fewer patients (33–56%) for the purpose of social reinforcement, which is consistent with the results of this study. The hypothalamic pituitary adrenal axis (HPA) participates in the affect regulation, and adolescence is the key period of HPA axis development. The limbic system and the prefrontal lobe that regulate the HPA axis undergo obvious developmental changes, and the sensitivity of the HPA axis to stress is prone to change. Patients with NSSI have passive HPA axis response under pressure, poor emotional regulation ability, and are prone to impulsive behavior (46).

Furthermore, we compared the functions of NSSI among different characteristic populations. Gender cannot affect the functional score of NSSI, while the frequency of NSSI had an effect. Patients with repeated NSSI supported significantly higher functional scores (except social influence). Combined with our findings that the behavioral addiction score of repeated NSSI was twice than that of occasional NSSI, we can infer that NSSI will increase addiction and strengthen various functions after repeated occurrence. Previous studies have also provided evidence for this point (47). The study also found that there were differences in the functional scores among different mental diseases. Therefore, we further explored the distribution characteristics of NSSI functions and found that NSSI patients can be divided into five functional subtypes. There was an interaction between functional subtypes and mental diseases in patients with repeated NSSI.

Specifically, the most supportive functional subtype in depression was “Subtype II-Sensation Seeking.” As is known to all, anhedonia is defined as loss of interest or pleasure in usual activities and is designated as one of two essential features of MDD, which is related to abnormal reward processing in the brain (48). The ventral striatum and orbitofrontal cortex (OFC) are related to pleasure experience, and the subjective evaluation of pleasure is also related to the orbitofrontal cortex. The ventral striatum and OFC activities of MDD individuals with anhedonia were reduced (49). Recent fMRI research findings based on the gambling task paradigm found that there was higher activation of the OFC when patients with NSSI following an unexpected reward. The function of “sensation seeking” is usually interpreted as seeking stimulation and experiencing excitement (50). Whether the “sensation seeking” function of NSSI can be used as a self-compensation for “anhedonia” in MDD patients, needs further research. Besides, it’s worth noting that the function of emotion regulation in this subtype also played a prominent role. In other words, patients with functional subtype II often engaged in NSSI for reasons of sensation seeking and emotion regulation. Sensation seeking meant patients inducing a positive state through NSSI, which was a specific function of intra-personal emotion regulation (2). For patients with depressive disorder who most support this functional subtype, the improvement of emotional regulation ability is the most critical intervention and Dialectical Behavior Therapy (DBT) or Emotional Regulation Group Therapy (ERGT) (51) might be particularly helpful.

Among patients with BP, the most functional subtype of NSSI was “Subtype IV-Anti-Suicide.” Self-injury may be considered as a way to express suicidal thoughts without risk of death. It is an alternative or compromise to suicide desire. At the same time, it is well known that BP has the highest suicide rate of all mental diseases (52). Although the NSSI function of “Anti-Suicide” was the most significant in this group, NSSI was also a risk factor for suicide attempt (OR = 4.27) (9). This relationship can be explained by the interpersonal theory of suicide. Even though NSSI can cope with suicide tendency in the short term, repeated NSSI can enhance the individual’s ability to self-harm and ultimately increase the suicide risk (9, 30, 37). Previous study showed that the anti-suicide function significantly predicts the duration of current suicidal ideation (53). Therefore, it is significant to take active intervention for NSSI toward patients with BP to reduce their suicide risk and it is also necessary to regularly evaluate suicide ideation.

Patients with ED supported the “Subtype III-Social Influence” most. The study found that the function of strengthening ED behavior overlap with functions of NSSI, while social function is more relevant to ED behavior (54, 55). Linked to the results of this study, when patients with ED combined with NSSI, the “social influence” function was more significant than in other patients with NSSI. This also reflects those patients with ED had acuter interpersonal problems, suggesting that we should pay attention to the teaching of interpersonal and communication skills, and encourage patients to adopt more appropriate coping methods rather than ED behavior or NSSI.

Finally, “Typical-NSSI” and “Untypical-NSSI” were two completely different subtypes. Patients with “Typical-NSSI” had high score of the four functions and NSSI might be the usual way that patients cope with various stresses. Whether the relief of negative emotion or arousal of positive emotion, or the sense of control obtained in interpersonal aspects, these functions would reinforce NSSI behaviors and become a more automatic and conditional response to stresses over time. This might lead to an increase in the frequency and severity of NSSI, or patients need more frequent and stronger NSSI to achieve the initial effect, that is, NSSI addiction (56). Previous study showed that there was a significant positive correlation between NSSI functional score and addictive features (57). Patients with “Typical-NSSI” might have a higher risk of addiction to NSSI. Attention should be paid to the evaluation and intervention of addictive features, and reduce the thoughts or impulses of NSSI, as well as teach emotional regulation skills and interpersonal communication skills, in order to replace the NSSI functions and reduce its addiction. As for the “Untypical NSSI,” present study found that most patients with occasional NSSI belonged to this subtype. Patients with “Untypical NSSI” might occasionally choose NSSI as their coping way when experiencing stresses. However, it should be warned that NSSI may disappear or become more serious as time goes by Daukantaitë et al. (58), Manca el al. (59). For those patients, we should pay attention to treatment of NSSI, meanwhile, follow-up and outcome are also important.

Based on the above findings, the person-centered specific intervention is the key in clinical practice. Dialectical Behavior Therapy (DBT) for NSSI has been encouraged which is a treatment involving individual and group modes developed by Marsha Linehan. For patients with different NSSI functional subtypes, focusing on teaching different DBT skills is expected to effectively reduce NSSI, reduce the waste of human resources and improve the efficiency of intervention.

## 5. Limitations

This study has several limitations. First, all participants were in treatment in a psychiatric service and the results may not be generalizable to a community sample. Second, the data included retrospective self-report which may be inaccurate due to recall bias, and follow-up after discharge can improve the accuracy of data. Third, the Chinese version of OSI-F revised by Zhang et al. (22) was matched with the original version developed in 2003 which not was revised in the original language. We explored the structure of the Chinese version of OSI-F and used the results of exploratory factor analysis to evaluate and analyze the NSSI function. Although the factor structure is supported by more theoretical models, it still needs to be verified by independent samples in the later stage. We also conducted functional analysis by adopting items that overlapped with the revised English edition. The relevant results are shown in Supplementary material.

## 6. Conclusion

In general, the current results showed that NSSI is more common in mental disorder groups, and the severity is worrying with higher frequency, more areas injured, more methods taken, and severer addiction. Individuals with more risk factors are more likely to engage in repeated NSSI. Unfavorable internal personal characteristics are important risk factors for the occurrence of NSSI, while unfavorable external environmental factors will promote these patients to have more serious NSSI behavior. It enlightens us to pay special attention to mental disorder groups with unfavorable internal personal and unfavorable external environmental characteristics, to early identify high-risk groups and take a targeted preventive intervention. Equally important and advanced finding is that NSSI patients have different functional subtypes which interact with the diagnosis of mental disorders. When treating NSSI patients with different mental disorders, it is very meaningful to take person-centered measures in combination with the subtypes.

## Data availability statement

The raw data supporting the conclusions of this article will be made available by the authors, without undue reservation.

## Ethics statement

The studies involving human participants were reviewed and approved by the Biomedical Ethics Committee of the Sixth Hospital of Peking University. Written informed consent to participate in this study was provided by the participant himself or his legal guardian/next of kin.

## Author contributions

HuY: conceptualization, methodology, investigation, writing – original draft, and formal analysis. YZ and ZL: methodology, formal analysis, and writing – revision of the manuscript. ML and YG: methodology and investigation. DM and ZK: writing – revision of the manuscript. YS: methodology and writing – revision of the manuscript. QL, HaY, LY, PS, CS, SS, and WY: conceptualization, methodology, investigation, writing – revision of the manuscript, support, and supervision. All authors contributed to the article and approved the submitted version.

## References

[1] Nock M Joiner T Jr Gordon K Lloyd-Richardson E Prinstein M. Non-suicidal self-injury among adolescents: diagnostic correlates and relation to suicide attempts. *Psychiatry Res.* (2006) 144:65–72. 10.1016/j.psychres.2006.05.010 16887199

[2] Taylor P Jomar K Dhingra K Forrester R Shahmalak U Dickson J. A meta-analysis of the prevalence of different functions of non-suicidal self-injury. *J Affect Disord.* (2018) 227:759–69. 10.1016/j.jad.2017.11.073 29689691

[3] Swannell S Martin G Page A Hasking P St John N. Prevalence of nonsuicidal self-injury in nonclinical samples: systematic review, meta-analysis and meta-regression. *Suicide Life Threat Behav.* (2014) 44:273–303. 10.1111/sltb.12070 24422986

[4] Mercado M Holland K Leemis R Stone D Wang J. Trends in emergency department visits for nonfatal self-inflicted injuries among youth aged 10 to 24 years in the United States, 2001-2015. *JAMA.* (2017) 318:1931–3. 10.1001/jama.2017.13317 29164246 PMC5753998

[5] Peterson C Xu L Leemis R Stone D. Repeat self-inflicted injury among U.S. Youth in a large medical claims database. *Am J Prev Med.* (2019) 56:411–9. 10.1016/j.amepre.2018.09.009 30658863 PMC6380925

[6] Lin M Li P Lu Q. Research progress of non suicidal self injury in adolescents. *J Psychiatry.* (2018) 31:67–70.

[7] Mars B Heron J Crane C Hawton K Lewis G Macleod J Clinical and social outcomes of adolescent self harm: population based birth cohort study. *BMJ.* (2014) 349:g5954. 10.1136/bmj.g5954 25335825 PMC4205277

[8] Wilkinson P Kelvin R Roberts C Dubicka B Goodyer I. Clinical and psychosocial predictors of suicide attempts and nonsuicidal self-injury in the adolescent depression antidepressants and psychotherapy trial (ADAPT). *Am J Psychiatry.* (2011) 168:495–501. 10.1176/appi.ajp.2010.10050718 21285141

[9] Ribeiro J Franklin J Fox K Bentley K Kleiman E Chang B Self-injurious thoughts and behaviors as risk factors for future suicide ideation, attempts, and death: a meta-analysis of longitudinal studies. *Psychol Med.* (2016) 46:225–36. 10.1017/s0033291715001804 26370729 PMC4774896

[10] Barrocas A Jenness J Davis T Oppenheimer C Technow J Gulley L Developmental perspectives on vulnerability to nonsuicidal self-injury in youth. *Adv Child Dev Behav.* (2011) 40:301–36. 10.1016/b978-0-12-386491-8.00008-6 21887965

[11] Plener P Kaess M Schmahl C Pollak S Fegert J Brown R. Nonsuicidal self-injury in adolescents. *Dtsch Arztebl Int.* (2018) 115:23–30. 10.3238/arztebl.2018.0023 29366448 PMC5787659

[12] Singhal A Ross J Seminog O Hawton K Goldacre M. Risk of self-harm and suicide in people with specific psychiatric and physical disorders: comparisons between disorders using English national record linkage. *J R Soc Med.* (2014) 107:194–204. 10.1177/0141076814522033 24526464 PMC4023515

[13] Esposito-Smythers C Goldstein T Birmaher B Goldstein B Hunt J Ryan N Clinical and psychosocial correlates of non-suicidal self-injury within a sample of children and adolescents with bipolar disorder. *J Affect Disord.* (2010) 125:89–97. 10.1016/j.jad.2009.12.029 20089313 PMC2888943

[14] MacPherson H Weinstein S West A. Non-suicidal self-injury in pediatric bipolar disorder: clinical correlates and impact on psychosocial treatment outcomes. *J Abnorm Child Psychol.* (2018) 46:857–70. 10.1007/s10802-017-0331-4 28725956

[15] Zubrick S Hafekost J Johnson S Sawyer M Patton G Lawrence D. The continuity and duration of depression and its relationship to non-suicidal self-harm and suicidal ideation and behavior in adolescents 12-17. *J Affect Disord.* (2017) 220:49–56. 10.1016/j.jad.2017.05.050 28595098

[16] Cucchi A Ryan D Konstantakopoulos G Stroumpa S Kaçar A Renshaw S Lifetime prevalence of non-suicidal self-injury in patients with eating disorders: a systematic review and meta-analysis. *Psychol Med.* (2016) 46:1345–58. 10.1017/s0033291716000027 26954514

[17] Chai Y Luo H Wong G Tang J Lam T Wong I Risk of self-harm after the diagnosis of psychiatric disorders in Hong Kong, 2000-10: a nested case-control study. *Lancet Psychiatry.* (2020) 7:135–47. 10.1016/s2215-0366(20)30004-3 31974072

[18] Fox K Franklin J Ribeiro J Kleiman E Bentley K Nock M. Meta-analysis of risk factors for nonsuicidal self-injury. *Clin Psychol Rev.* (2015) 42:156–67. 10.1016/j.cpr.2015.09.002 26416295 PMC4772426

[19] Jiang Y You J Hou Y Du C Lin M Zheng X Buffering the effects of peer victimization on adolescent non-suicidal self-injury: the role of self-compassion and family cohesion. *J Adolesc.* (2016) 53:107–15. 10.1016/j.adolescence.2016.09.005 27710776

[20] Klonsky ED. The functions of deliberate self-injury: a review of the evidence. *Clin Psychol Rev.* (2007) 27:226–39. 10.1016/j.cpr.2006.08.002 17014942

[21] Singhal N Bhola P Reddi V Bhaskarapillai B Joseph S. Non-suicidal self-injury (NSSI) among emerging adults: sub-group profiles and their clinical relevance. *Psychiatry Res.* (2021) 300:113877. 10.1016/j.psychres.2021.113877 33831810

[22] Zhang F Cheng W Zp X Wj L. Study on reliability and validity of Chinese version of Ottawa self-injury inventory. *J Shanghai Jiaotong Univ.* (2015) 35:460–4.

[23] Wang X. *Handbook of Mental Health Assessment Scales.* Beijing: Chinese Mental Health Journal (1999). p. 31–5.

[24] Pervin L John O Huang X. *Handbook of Personality Theory and Research.* Shanghai: East China Normal University Press (2003).

[25] Bai Y Fan F. A study on the Internet dependence of college students: the revising and applying of a measurement. *Psychol Dev Educ.* (2005) 4:99–104.

[26] Zhao X Zhang Y Li L Zhou Y. Evaluation on reliability and validity of Chinese version of childhood trauma questionnaire. *Chin J Clin Rehabil.* (2005) 16:209–11.

[27] Lopez Garcia M Garcia-Rodenas R Gonzalez Gomez A. K-means algorithms for functional data. *Neurocomputing.* (2015) 151:231–45. 10.1016/j.neucom.2014.09.048

[28] Glenn C Klonsky E. Nonsuicidal self-injury disorder: an empirical investigation in adolescent psychiatric patients. *J Clin Child Adolesc Psychol.* (2013) 42:496–507. 10.1080/15374416.2013.794699 23682597 PMC4433043

[29] Westers N Culyba A. Nonsuicidal self-injury: a neglected public health problem among adolescents. *Am J Public Health.* (2018) 108:981–3. 10.2105/ajph.2018.304550 29995480 PMC6050854

[30] Joiner T Ribeiro J Silva C. Nonsuicidal self-injury, suicidal behavior, and their co-occurrence as viewed through the lens of the interpersonal theory of suicide. *Curr Dir Psychol Sci.* (2012) 21:342–7. 10.1177/0963721412454873

[31] Liu Z Tein J Jia C Liu X. Depression as a mediator between frequent nightmares and non-suicidal self-injury among adolescents: a 3-wave longitudinal model. *Sleep Med.* (2021) 77:29–34. 10.1016/j.sleep.2020.11.015 33307303

[32] Peters E Bowen R Balbuena L. Mood instability contributes to impulsivity, non-suicidal self-injury, and binge eating/purging in people with anxiety disorders. *Psychol Psychother.* (2019) 92:422–38. 10.1111/papt.12192 30003688

[33] Nock M. Self-injury. *Annu Rev Clin Psychol.* (2010) 6:339–63. 10.1146/annurev.clinpsy.121208.131258 20192787

[34] Nock M Prinstein MJ. A functional approach to the assessment of self-mutilative behavior. *J Consult Clin Psychol.* (2004) 72:885–90. 10.1037/0022-006x.72.5.885 15482046

[35] Lê-Scherban F Brenner A Schoeni R. Childhood family wealth and mental health in a national cohort of young adults. *SSM Popul Health.* (2016) 2:798–806. 10.1016/j.ssmph.2016.10.008 28584861 PMC5455782

[36] Pascoe J Wood D Duffee J Kuo A. Mediators and adverse effects of child poverty in the United States. *Pediatrics.* (2016) 137:e20160340. 10.1542/peds.2016-0340 26962239

[37] Kaess M Eppelmann L Brunner R Parzer P Resch F Carli V Life events predicting the first onset of adolescent direct self-injurious behavior-a prospective multicenter Study. *J Adolesc Health.* (2020) 66:195–201. 10.1016/j.jadohealth.2019.08.018 31677986

[38] Kelada L Hasking P Melvin G. The relationship between nonsuicidal self-injury and family functioning: adolescent and parent perspectives. *J Marital Fam Ther.* (2016) 42:536–49. 10.1111/jmft.12150 26725333

[39] Wang Y Li X Ng C Xu D Hu S Yuan T. Risk factors for non-suicidal self-injury (NSSI) in adolescents: a meta-analysis. *EClinicalMedicine.* (2022) 46:101350. 10.1016/j.eclinm.2022.101350 35330803 PMC8938878

[40] Goddard A Hasking P Claes L McEvoy P. Big five personality clusters in relation to nonsuicidal self-injury. *Arch Suicide Res.* (2021) 25:390–405. 10.1080/13811118.2019.1691099 31769355

[41] Mészáros G Gyõri D Horváth L Szentiványi D Balázs J. Nonsuicidal self-injury: its associations with pathological internet use and psychopathology among adolescents. *Front Psychiatry.* (2020) 11:814. 10.3389/fpsyt.2020.00814 32922320 PMC7456921

[42] Tang J Ma Y Lewis S Chen R Clifford A Ammerman B Association of internet addiction with nonsuicidal self-injury among adolescents in China. *JAMA Netw Open.* (2020) 3:e206863. 10.1001/jamanetworkopen.2020.6863 32496567 PMC7273191

[43] Monroe S Simons A. Diathesis-stress theories in the context of life stress research: implications for the depressive disorders. *Psychol Bull.* (1991) 110:406–25. 10.1037/0033-2909.110.3.406 1758917

[44] Vergara G Stewart J Cosby E Lincoln S Auerbach R. Non-Suicidal self-injury and suicide in depressed adolescents: impact of peer victimization and bullying. *J Affect Disord.* (2019) 245:744–9. 10.1016/j.jad.2018.11.084 30448758 PMC6351200

[45] Tatnell R Kelada L Hasking P Martin G. Longitudinal analysis of adolescent NSSI: the role of intrapersonal and interpersonal factors. *J Abnorm Child Psychol.* (2014) 42:885–96. 10.1007/s10802-013-9837-6 24343795

[46] Klimes-Dougan B Begnel E Almy B Thai M Schreiner M Cullen K. Hypothalamic-pituitary-adrenal axis dysregulation in depressed adolescents with non-suicidal self-injury. *Psychoneuroendocrinology.* (2019) 102:216–24. 10.1016/j.psyneuen.2018.11.004 30590339

[47] Davis S Lewis C. Addiction to self-harm? The case of online postings on self-harm message boards. *Int J Ment Health Addict.* (2018) 17:1020–35. 10.1007/s11469-018-9975-8

[48] Der-Avakian A Markou A. The neurobiology of anhedonia and other reward-related deficits. *Trends Neurosci.* (2012) 35:68–77. 10.1016/j.tins.2011.11.005 22177980 PMC3253139

[49] Keedwell P Andrew C Williams S Brammer M Phillips M. The neural correlates of anhedonia in major depressive disorder. *Biol Psychiatry.* (2005) 58:843–53. 16043128 10.1016/j.biopsych.2005.05.019

[50] Martin J Cloutier P Levesque C Bureau J Lafontaine M Nixon M. Psychometric properties of the functions and addictive features scales of the Ottawa self-injury inventory: a preliminary investigation using a university sample. *Psychol Assess.* (2013) 25:1013–8. 10.1037/a0032575 23647037

[51] Andover M Morris B. Expanding and clarifying the role of emotion regulation in nonsuicidal self-injury/développer et clarifier le rôle de la régulation émotionnelle dans l’automutilation non suicidaire. *Can J Psychiatry.* (2014) 59:569. 10.1177/070674371405901102 PMC4244875 25565472

[52] Miller J Black D. Bipolar disorder and suicide: a review. *Curr Psychiatry Rep.* (2020) 22:6. 10.1007/s11920-020-1130-0 31955273

[53] Kraus L Schmid M In-Albon T. Anti-suicide function of nonsuicidal self-injury in female inpatient adolescents. *Front Psychiatry.* (2020) 11:490. 10.3389/fpsyt.2020.00490 PMC7283589 32581870

[54] Muehlenkamp J Takakuni S Brausch A Peyerl N. Behavioral functions underlying NSSI and eating disorder behaviors. *J Clin Psychol.* (2019) 75:1219–32. 30672588 10.1002/jclp.22745

[55] Wedig M. *Psychological Meanings and Functions of Non-Suicidal Self-Injury and Eating Disorders.* Berlin: Springer Berlin Heidelberg (2014). p. 73–84.

[56] Chen H Zhou J. Research progress on the addictive characteristics of non-suicidal self-injury. *Chin J Psychiatry.* (2022) 55:64–8.

[57] Nixon M Levesque C Preyde M Vanderkooy J Cloutier P. The Ottawa self-injury inventory: evaluation of an assessment measure of nonsuicidal self-injury in an inpatient sample of adolescents. *Child Adolesc Psychiatry Ment Health.* (2015) 9:26. 10.1186/s13034-015-0056-5 26157482 PMC4495629

[58] Daukantaitë D Lundh L Wångby-Lundh M Claréus B Bjärehed J Zhou Y What happens to young adults who have engaged in self-injurious behavior as adolescents? A 10-year follow-up. *Eur Child Adolesc Psychiatry.* (2021) 30:475–92. 10.1007/s00787-020-01533-4 32318877 PMC8019412

[59] Manca M Presaghi F Cerutti R. Clinical specificity of acute versus chronic self-injury: measurement and evaluation of repetitive non-suicidal self-injury. *Psychiatry Res.* (2014) 215:111–9. 10.1016/j.psychres.2013.10.010 24210667

