# Supplementary material for: High-risk group and functional subtypes of non-suicidal self-injury in young adults with mental disorders
Source: Front Psychiatry. 2023 Feb 23;14:1098178. doi: 10.3389/fpsyt.2023.1098178 (PMC9996010; doi:10.3389/fpsyt.2023.1098178)
Supplement: Supplementary file 2 [file Data_Sheet_1.docx]

Supplementary Material

# Factor structure of OSI-F revised by Zhang et al.

This study conducted an item analysis of the Ottawa self-injury inventory-function (OSI-F). The correlation between each item and the total score was calculated. The results showed that all correlation coefficients *r*>0.3, and *p*<0.001. Therefore, all items were retained for exploratory factor analysis (EFA). Bartlett's spherical test showed that the scale was suitable for factor analysis (*χ2*=2519.562, *p*<0.001, Kaiser Meyer Olkin=0.852). In the EFA, principal component analysis and maximum variance orthogonal rotation method were used and we extracted 6 common factors with eigenvalue > 1, and the cumulative variance was 59.638%. Removed 2 items with a load of less than 0.45 (“To punish myself”, “To satisfy voices inside or outside me telling me to do it”) and do EFA again. Extract 6 common factors with an eigenvalue > 1, and the cumulative variance is 60.687%. The factor loadings of item for each obtained factor were all above 0.45 (see Supplementary Table 6). Factor 1 was named emotion regulation (1, 8, 10, 13, 17, 19, 21, 26), factor 3 was named sensation seeking (2, 4, 7, 25, 28), and factor 5 was named anti-suicide (23, 24). According to the content and clinical interpretation of the items included in factor 2, factor 4 and factor 6, they all belonged to the social influence function(Martin et al.), so these three factors were combined into one social influence factor (3, 5, 9, 11, 12, 14, 15, 16, 20, 22, 27, 29). To sum up, OSI-F evaluates the four functions of NSSI, which are “emotion regulation”, “sensation seeking”, “social influence” and “anti-suicide”, consists of 27 items.

| **Table 6 Factor Loadings, Eigenvalues for the Function Factors of the OSI-F-** **revised by Zhang et al.** | | | | | | | |
| --- | --- | --- | --- | --- | --- | --- | --- |
| **Items** | **Factor1** | **Factor 2** | **Factor 3** | **Factor 4** | **Factor 5** | **Factor 6** | **Communalities** |
|  | **Emotion regulation** | **Social influence** | **Sensation seeking** | **Social influence** | **Anti-suicide** | **Social influence** |  |
| 21 To relieve feelings of sadness or feeling "down" | 0.764 |  |  |  |  |  | 0.646 |
| 17 To help me escape from uncomfortable feelings or moods | 0.750 |  |  |  |  |  | 0.613 |
| 1 To release unbearable tension | 0.735 |  |  |  |  |  | 0.544 |
| 8 To relieve nervousness/fearfulness | 0.643 |  |  |  |  |  | 0.603 |
| 10 To distract me from unpleasant memories | 0.641 |  |  |  |  |  | 0.501 |
| 19 To experience physical pain in one area, when the other pain I feel is unbearable | 0.536 |  |  |  |  |  | 0.421 |
| 13 To release anger | 0.530 |  |  |  |  |  | 0.432 |
| 26 To release frustration | 0.507 |  |  |  |  |  | 0.61 |
| 27 To get out of doing something that I don't want to do |  | 0.791 |  |  |  |  | 0.73 |
| 20 To stop people from expecting so much from me |  | 0.696 |  |  |  |  | 0.596 |
| 9 To avoid getting in trouble for something I did |  | 0.672 |  |  |  |  | 0.602 |
| 22 To have control in a situation where no one can influence me |  | 0.466 |  |  |  |  | 0.493 |
| 2 To experience a "high" |  |  | 0.833 |  |  |  | 0.733 |
| 7 To provide a sense of excitement that feels exhilarating |  |  | 0.824 |  |  |  | 0.774 |
| 28 For no reason that I know about, it just happens sometimes |  |  | 0.557 |  |  |  | 0.387 |
| 25 To produce a sense of being real when I feel numb and "unreal" |  |  | 0.514 |  |  |  | 0.604 |
| 4 To stop feeling alone and empty |  |  | 0.498 |  |  |  | 0.558 |
| 5 To get care and attention from other people |  |  |  | 0.791 |  |  | 0.711 |
| 15 To show others how hurt or damaged I am |  |  |  | 0.753 |  |  | 0.637 |
| 14 To stop my friends/boyfriend/girlfriend from being angry with me |  |  |  | 0.615 |  |  | 0.537 |
| 12 To belong to a group |  |  |  | 0.532 |  |  | 0.529 |
| 3 To stop my parents from being angry at me |  |  |  | 0.465 |  |  | 0.531 |
| 23 To stop me from thinking about ideas of killing myself |  |  |  |  | 0.913 |  | 0.879 |
| 24 To stop me from acting out ideas of killing myself |  |  |  |  | 0.911 |  | 0.882 |
| 11 To change my body image and/or appearance |  |  |  |  |  | 0.716 | 0.623 |
| 29 To prove to myself how much I can take |  |  |  |  |  | 0.713 | 0.618 |
| 16 To show others how strong or tough I am |  |  |  |  |  | 0.580 | 0.459 |
| **Eigenvalues** | 7.330 | 2.916 | 1.967 | 1.769 | 1.208 | 1.063 |  |
| **Variance%** | 14.523 | 11.005 | 9.852 | 8.863 | 8.126 | 7.827 |  |

| **Table 7 Factor Loadings, Eigenvalues for the Function Factors of the OSI-F (revised English edition)** | | | | |
| --- | --- | --- | --- | --- |
| **Items** | **Factor1** | **Factor 2** | **Factor 3** | **Factor 4** |
|  | **Emotion regulation** | **Social influence** | **Sensation seeking** | **Social influence** |
| 19 To experience physical pain in one area, when the other pain I feel is unbearable | 0.697 |  |  |  |
| 21 To relieve feelings of sadness or feeling "down" | 0.676 |  |  |  |
| 25 To produce a sense of being real when I feel numb and "unreal" | 0.642 |  |  |  |
| 10 To distract me from unpleasant memories | 0.623 |  |  |  |
| 1 To release unbearable tension | 0.602 |  |  |  |
| 13 To release anger | 0.597 |  |  |  |
| 26 To release frustration | 0.582 |  |  |  |
| 23 To stop me from thinking about ideas of killing myself | 0.543 |  |  |  |
| 6 To punish myself | 0.499 |  |  |  |
| 27 To get out of doing something that I don't want to do |  | 0.774 |  |  |
| 9 To avoid getting in trouble for something I did |  | 0.65 |  |  |
| 3 To stop my parents from being angry at me |  | 0.649 |  |  |
| 20 To stop people from expecting so much from me |  | 0.627 |  |  |
| 15 To show others how hurt or damaged I am |  | 0.471 |  |  |
| 2 To experience a "high" |  |  | 0.867 |  |
| 7 To provide a sense of excitement that feels exhilarating |  |  | 0.846 |  |
| 4 To stop feeling alone and empty |  |  | 0.492 |  |
| 29 To prove to myself how much I can take |  |  | 0.465 |  |
| 12 To belong to a group |  |  |  | 0.749 |
| 11 To change my body image and/or appearance |  |  |  | 0.693 |
| **Eigenvalues** | 5.92 | 2.18 | 1.543 | 1.377 |
| **Variance%** | 18.93 | 14.584 | 12.126 | 9.456 |

# Factor structure of OSI-F-revised English edition

We choose twenty items from the Chinese version revised by Zhang et al. that overlap with the English edition revised in 2015(Nixon et al.). Then we conducted the confirmatory factor analysis (CFA) through the Amos 26.0 to test and verify the factor structure of the English edition revised in 2015 in our samples. The model was composed of four factors (Internal Emotion Regulation-IER, Social Influence-SI, External Emotion Regulation-EER, and Sensation Seeking-SS). The fit of the model was deemed inadequate (χ^2^/df=1.93; RMSEA=0.070; CFI=0.860; GFI=0.795) (Figure S1). The results indicated that this model was not applicable to the sample in current study.

Next, we further conducted the EFA with the items overlap with the English edition revised in 2015. Bartlett's spherical test showed that the scale was suitable for factor analysis (*χ2*=1333.196, *p*<0.001, Kaiser Meyer Olkin=0.846). The principal component analysis and maximum variance orthogonal rotation method were used and we extracted 4 common factors with eigenvalue > 1, and the cumulative variance was 55.096%. The factor loadings of item for each obtained factor were all above 0.45 (see Supplementary Table 7). Factor 1 was named emotion regulation (1, 6, 10, 13, 19, 21,23, 25, 26) and factor 3 was named sensation seeking (2, 4, 7, 29). According to the content and clinical interpretation of the items included in factor 2 and factor 4, they all belonged to the social influence function(Martin et al.). The only item represented the function of anti-suicide (“To stop me from thinking about ideas of killing myself”) belonged to the factor1. The factor structure according to the results of EFA showed that the functions of NSSI in current sample was different from that in revised English edition.

The possible reasons for the differences were diverse culture, ethnic, and environments, etc. Eventually, we chose the OSI-F revised by Zhang et al. and analyzed the NSSI function based on factor structure that we explored among sample of current study (see Supplementary Table 6).


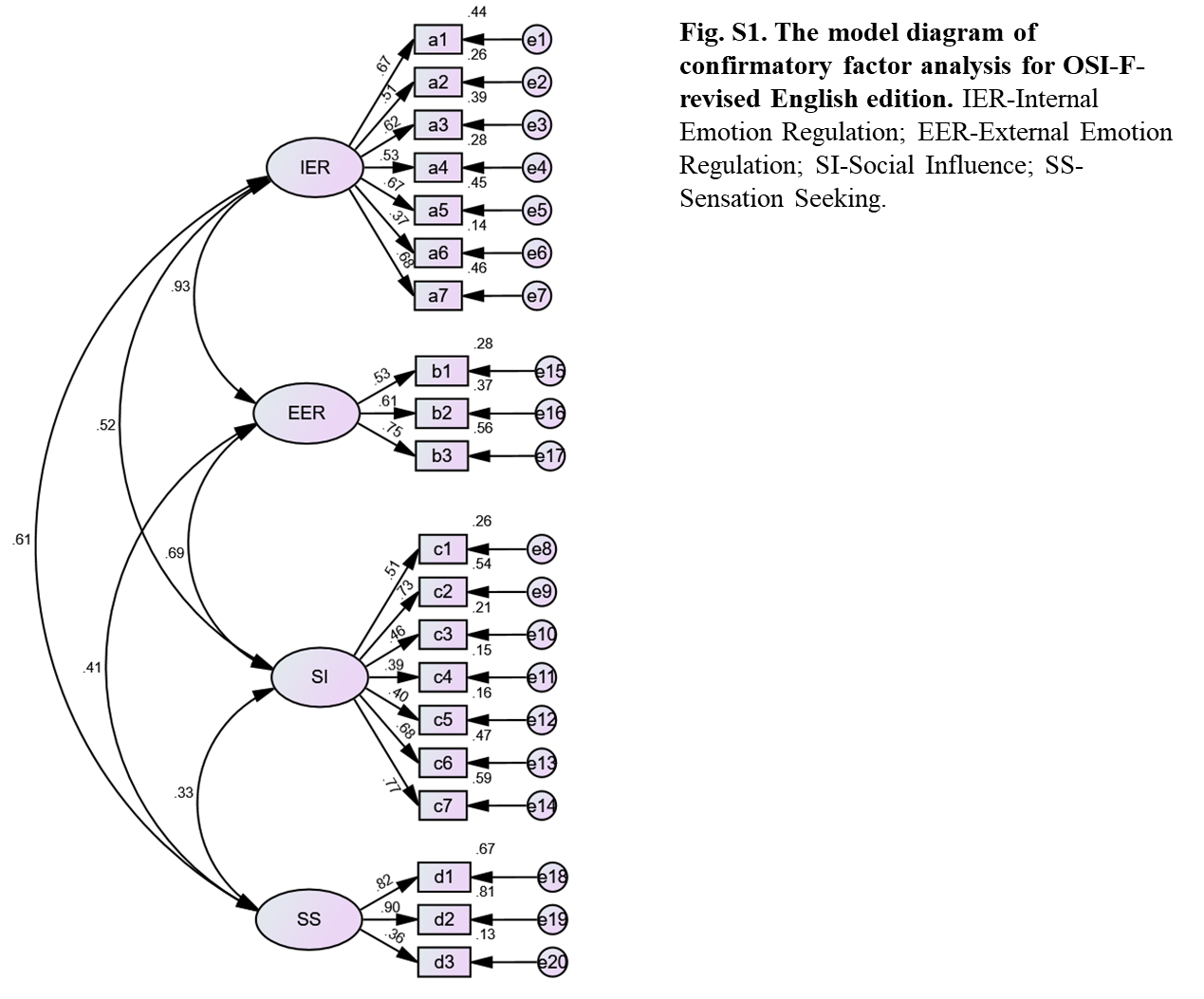


**Supplemental References**

Martin, J., P. F. Cloutier, C. Levesque, J. F. Bureau, M. F. Lafontaine, and M. K. Nixon. 2013. "Psychometric properties of the functions and addictive features scales of the Ottawa Self-Injury Inventory: a preliminary investigation using a university sample." *Psychol Assess* 25 (3):1013-8. doi: 10.1037/a0032575.

Nixon, M. K., C. Levesque, M. Preyde, J. Vanderkooy, and P. F. Cloutier. 2015. "The Ottawa Self-Injury Inventory: Evaluation of an assessment measure of nonsuicidal self-injury in an inpatient sample of adolescents." *Child Adolesc Psychiatry Ment Health* 9:26. doi: 10.1186/s13034-015-0056-5.
